# Near-infrared fluorescence imaging-guided focused ultrasound-mediated therapy against Rheumatoid Arthritis by MTX-ICG-loaded iRGD-modified echogenic liposomes

**DOI:** 10.7150/thno.44865

**Published:** 2020-08-08

**Authors:** Haohan Wu, Yanni He, Hao Wu, Meijun Zhou, Zhili Xu, Ran Xiong, Fei Yan, Hongmei Liu

**Affiliations:** 1 The Second School of Clinical Medicine, Southern Medical University, Guangzhou 510515, China.; 2 Department of Ultrasound, Institute of Ultrasound in Musculoskeletal Sports Medicine, Guangdong Second Provincial General Hospital, Guangzhou 510317, China.; 3 CAS Key Laboratory of Quantitative Engineering Biology, Shenzhen Institute of Synthetic Biology, Shenzhen Institutes of Advanced Technology, Chinese Academy of Sciences, Shenzhen 518055, China.

**Keywords:** Echogenic liposomes, Rheumatoid arthritis, Ultrasound-controlled drug release, Near-infrared fluorescence imaging, iRGD

## Abstract

Rheumatoid arthritis (RA), a common inflammatory disorder of the joints characterized by synovitis and pannus formation, often results in irreversible joint erosion and disability. Methotrexate (MTX) is the first-line drug against RA, but the therapeutic effects are sub-optimal due to its poor retention at the target sites and systemic side effects. Multifunctional nanoparticles are highly promising agents for minimally invasive, traceable and effective targeted therapy.

**Methods:** This study developed iRGD peptide-functionalized echogenic liposomes (iELPs) which encapsulates MTX and indocyanine green (ICG) fluorescent probe through the thin film-hydration method.

**Results:** The resulting iELPs showed high affinity for endothelial cells overexpressing αvβ3 integrin, favorable acoustic response and fluorescence tracking properties. Also, near-infrared (NIR) fluorescence imaging of iELPs and ultrasound-triggered drug release of MTX were proved in a mouse RA model, greatly improving the therapeutic efficacy and reducing MTX side effects. Histological assessment of the articular tissues further revealed significantly lower inflammatory cell infiltration and angiogenesis in the iELPs-treated and sonicated mice.

**Conclusion:** Our study provides a promising nanoplatform for integrating ultrasound-controlled drug release and NIR fluorescence imaging for RA treatment.

## Introduction

Rheumatoid arthritis (RA) is a chronic autoimmune disease characterized by synovitis and pannus formation in multiple joints, which progressively erodes the joint and often leads to disability 1. Angiogenesis is an early event in joint inflammation that recruits activated monocytes and endothelial cells (ECs) into the synovium, which expands to form a pannus in the later stages of the disease 2. Methotrexate (MTX) is currently the first-line drug against RA 3, 4. However, it causes significant systemic toxicity such as liver damage, erosion of gastrointestinal mucosa and myelosuppression, which can lead to refractory RA 5, 6. Furthermore, oral or intravenous administration of the drug results in sub-optimal concentration at the target sites. Therefore, targeted accumulation of the drug at RA lesions can not only improve its therapeutic efficacy but also reduce the systemic side effects. Some researchers have achieved the above via intra-articular injection of MTX 7-9. For example, Nakaya et al. reported enhanced ultrasound (US)-triggered uptake of MTX into synovial cells following direct injection into the articular space, which significantly augmented its anti-inflammatory effects 10. However, repeated intra-articular injections are painful, and can increase the risk of infection and secondary tissue damage. Therefore, it is necessary to develop a minimally invasive, traceable and effective targeted therapy for RA.

Multifunctional nanoparticles are a highly promising platform for targeted and effective RA therapy in a minimally invasive manner. Alam et al. synthesized pH-responsive MTX-loaded nanoparticles that showed significantly greater therapeutic effect in a mouse RA model compared to the free drug 11. Lee et al. constructed heat-sensitive gold (Au) half-shell nanoparticles that could release the encapsulated MTX in a controlled manner when exposed to laser 12. However, laser-induced hyperthermia increases risk of periarticular soft tissue damage. In contrast, low-frequency ultrasound is a highly effective and relatively safe trigger for controlled drug release from nanoparticles. Kim et al. fabricated nitric oxide (NO)-loaded nanoparticles that released the gas at the subarachnoid hemorrhage lesions upon ultrasonic stimulation to therapeutically effective levels 13.

This study constructed MTX-loaded echogenic liposomes for targeted treatment of RA via low-frequency ultrasound-triggered drug release. During arthritic progression, inflammatory factors in the articular space stimulate the endothelial cells (ECs) and up-regulate integrin αvβ3 14. These liposomes were further decorated with the near-infrared (NIR) fluorescence probe indocyanine green (ICG) and the iRGD peptide which specifically binds to the αvβ3 integrin. The iELPs showed high affinity for endothelial cells, favorable acoustic response and fluorescence tracking *in vitro* and targeted accumulation, NIR fluorescence imaging and ultrasound-triggered controlled drug release in a mouse model of RA. It is therefore an effective nanoplatform for targeted therapy of RA.

## Materials and Methods

### Reagents

Dipalmitoylphosphatidylcholine (DPPC), 1,2-Distearoyl-sn-glycero-3-phosphoethanolamine-N-[met-hoxy (poly-ethylene glycol)-2000] (DSPE-PEG2000), 1,2-distearoylsn-glycero-3phosphoethanolamine-N-[maleimide(polyethylenegl-ycol)-2000] (DSPE-PEG2000-Mal) and cholesterol were purchased from Avanti Polar Lipids Inc (Alabaster, AL, USA). The iRGD peptide (CRGDKGPDC) was synthesized by GL Biochem Ltd. (Shanghai, China). Methotrexate (MTX) hydrate was purchased from Aladdin (Shanghai, China). Indocyanine green (ICG), tumor necrosis factor alpha (TNF-α) and '4, 6-diamidino-2-phenylindole (DAPI) were purchased from Sigma-Aldrich (St. Louis, MO, USA). Cell Counting Kit-8 (CCK-8) was obtained from Dojindo Laboratories (Tokyo, Japan). Calcein-AM, propidium iodide (PI) and 5-(and-6)-chloromethyl-2′,7′-dichlorodihydro-fluorescein diacetate, acetyl ester (CM-H2DCFDA) were purchased from Invitrogen (Eugene, Oregon, USA). Bovine type II collagen, complete Freund's adjuvant and incomplete Freund's adjuvant were purchased from Condrex (Redmond, WA, USA).

### Construction of echogenic liposomes

The iRGD-lipopeptide (DSPE-PEG2000-iRGD) was synthesized by coupling iRGD peptide to DSPE-PEG2000-maleimide via Michael-type addition reaction 15, 16. MTX and ICG were encapsulated into the liposomes by the thin film-hydration method, and the hydrated liposomes were lyophilized for 48 h using 5% sucrose as the cryoprotectant. The ensuing iRGD-MTX-ICG-ELIPs or iELPs were stored at 4°C in the dark. The MTX-ICG-ELIPs or ELPs (the echogenic liposomes containing MTX and ICG decorated) were similarly prepared minus the DSPE-PEG2000-iRGD.

### Characterization of echogenic liposomes

The average diameter, zeta potential and size distribution of the iELPs were determined by dynamic light scattering (Zetasizer Nano ZS, Malvern Instrument, UK), and their morphology and diameter were analyzed by transmission electron microscopy (TEM). The encapsulation efficiency (EE) of MTX and the amount released from the liposomes were determined by an UV/vis spectrometer (Lambda 25, Perkine Elmer, USA), calculated using the formula: EE% = (W_encap_ /W_total_) × 100%. The stability of liposomes was evaluated in terms of mean diameter and drug leakage. The photostability and imaging capability of free ICG and iELPs were assessed by fluorescent light exposure for 0, 6 and 12 h.

### Determination of ultrasonic response for iELPs

In order to evaluate the ultrasonic response of iELPs, *in vitro* high frequency ultrasound imaging was performed using the Vevo 2100 imaging system (VisualSonics, Canada). The iELPs were diluted 50-fold and imaged at different time points (0-4 h). Furthermore, the images of iELPs before and after ultrasonic irradiation (1 MHz, 0.2 MPa, 1 min, continuous-wave, sine wave) were obtained at 3 h after 100× dilution.

### Determination of drug release* in vitro*

The iELPs were irradiated with 1 MHz ultrasound at varying intensities and duration, loaded into dialysis bags (1k Da), and immersed in 30 ml phosphate buffer saline (PBS, pH 7.2). The solution was constantly stirred and replaced with fresh PBS after each sampling to maintain the volume. The amount of MTX released from the iELPs was measured at 304 nm by UV/vis spectrometer. Each sample was measured thrice.

### Cell lines and cell culture

The recipient human umbilical vein endothelial cells (HUVEC) were purchased from the American Type Culture Collection (ATCC). The effector human fibroblast-like synoviocyte line MH7A was obtained from Riken cell bank (Ibaraki, Japan). The RAW 264.7 mouse macrophages were obtained from the Cell Bank of Type Culture Collection of Chinese Academy of Sciences (Shanghai, China). The HUVECs and MH7A cells were cultured in Roswell Park Memorial Institute (RPMI) 1640 medium, and RAW 264.7 cells were cultured in Dulbecco's Modified Eagle Medium (DMEM) at 37°C under 5% CO_2_, respectively. The cells in the exponential growth phase with viability ≥ 98% were used for the experiments.

### Intracellular localization and specificity of iELPs

The expression levels of αvβ3 receptors in HUVECs, MH7A cells and RAW 264.7 cells were determined by Western blotting. HUVECs were incubated overnight with 1 ng/ml TNF-α to simulate the RA microenvironment, blocked with free iRGD for 30 minutes to prevent non-specific binding, and then treated with iELPs, ELPs or 2 µg ICG for 1h. After washing thrice with PBS, the cells were fixed with 4% paraformaldehyde for 20 min, stained with 10 μg/ml 4,6-diamidino-2-phenylindole (DAPI) for 5 min, and washed again with PBS. The cells were observed under a confocal laser scanning microscope (TCS SP5, Leica, Germany) at 63× magnification to track the intracellular localization of iELPs. Then the cells were harvested, and the fluorescence histograms of ICG were recorded by flow cytometer (BD Accuri C6, USA). RAW 264.7 and MH7A cells were similarly treated with iELPs and stained to determine the cell-type specificity of the iRGD-targeted liposomes.

### Analysis of cytotoxicity and apoptosis

MH7A cells were seeded in 96-well plates at the density of 1×10^4^/100 µl/well, and incubated for 24 h. After replacing the medium with fresh medium, the cells were irradiated with 1 MHz ultrasound at 0-0.3 MPa for 0-3 min and incubated further for 24 h. The viability of the cells was analyzed using the CCK-8 Kit. The absorbance at 450 nm was measured using a multimode plate reader (Synergy™4, BioTek, VT, USA). After standardizing the sonication parameters, the MH7A cells cultured for 24 h were incubated with PBS, free MTX, ELPs or iELPs for 3 h, washed once to remove the unbound liposomes, and irradiated with 1MHz ultrasound for 90 s. The treated cells were fixed with 4% paraformaldehyde, stained with calcein-AM and PI, and viewed with an inverted microscope (Olympus IX71, Japan) to identify the viable and dead cells.

HUVECs were seeded into the upper chambers of transwell inserts (24-well insert, pore size 0.4 μm) at the density of 1×10^5^ cells per well in complete RPMI-1640 medium with ELPs or iELPs. The lower chambers were seeded with 2×10^5^ MH7A cells in complete RPMI-1640 medium. After 24 h of incubation, washed once to remove the liposomes in the upper chambers, the established transwells were exposed to 1MHz ultrasound for 90 s, respectively. The viability of the MH7A cells was analyzed using the CCK-8 Kit.

### *In vivo* NIR fluorescence imaging and bio-distribution in main organs

Male DAB/1J mice were obtained from Vitalriver Experimental Animal Technology Ltd (Beijing, China), and maintained in accordance with the guidelines for the Care and Use of Laboratory Animals. All animal experiments were approved by Shenzhen Institutes of Advanced Technology, Chinese Academy of Sciences Animal Care and Use Committee. The collagen-induced arthritis (CIA) model was established using Type II collagen as previously described 17. The mice were monitored twice weekly for any signs of arthritis based on paw swelling and the clinical arthritis score on a scale of 0-4 17. Upon arthritic development (clinical score of 10-12), the joints were assessed by high-resolution Micro-computed tomography, hematoxylin and eosin (H&E) staining and immunohistochemical staining of vascular endothelial growth factor-A (VEGFA) and TNF-α.

Healthy murine ankle joints and small pieces of porcine tissue (1×1×1 cm) were sonicated with varying intensities and duration. The temperature was recorded using a thermal imager (Ti25, Fluke, USA). The sonicated tissues were dissected and photographed, and the parameters that resulted in no obvious macroscopic mechanical damage or hyperthermia were selected for the *in vivo* experiments.

To test the effectiveness of targeting by iELPs, the successfully modeled mice were randomized into three groups, and respectively injected with 100 µl iELPs, ELPs or free ICG (100 µg/ml ICG) via the tail vein. *In vivo* NIR fluorescence imaging was performed at 745 nm excitation and 840 nm emission using the Xenogen IVIS Spectrum system (PerkinElmer, Waltham, MA) at 30 min to 24 h after injection. In addition, some mice were sacrificed 24 h after injection, and their heart, liver, spleen, lung, kidney and paws were harvested. The organs were photographed to analyze the biodistribution of iELPs or ELPs using the *ex/in vivo* imaging system, and the fluorescence intensity ratios to that before or immediately after injection were calculated. Same methods were conducted in normal mice and CIA (the collagen-induced arthritis) model mice, to further confirm the accuracy drug delivery by iELPs.

To test the drug release by iELPs* in vivo*, the successfully modeled mice were injected with 100 µl iELPs (100 µg/ml ICG) via the tail vein. *In vivo* NIR fluorescence imaging was performed before and after ultrasonic irradiation, and the fluorescence intensity ratios were calculated.

### Targeted therapy *in vivo*

Once the clinical arthritis score reached 10-12, the mice were randomly divided into the control (G1), US (G2), MTX (G3), iELPs (G4), ELPs+US(G5) and iELPs+US (G6) groups (n = 6 each). The respective drugs were administered intravenously (MTX dose = 2.0 mg/kg), and the mice were irradiated at the arthritic joints with 0.2 MPa ultrasound for 3 min after the peak duration as measured. All treatments were repeated 7 days later for a total of four times. The mice were observed every other day for 28 days, and the clinical arthritis scores were recorded.

### Radiological and histological assessment of the ankle joints

One month after the suitable treatments, the micro-computed tomography (Micro-CT) 3D reconstruction of the ankle joints were taken using a high-resolution Micro-computed tomography (SkyScan 1176, BRUKER, GER). The mice were then sacrificed, and their hind paws were harvested and fixed in 4% formalin for 12 h. After decalcifying in 10% EDTA (Sigma) for 20 days at room temperature, 4 µm thick paraffin sections were cut and stained with H&E. The sections were examined for synovial inflammation and bone erosion.

### Statistical Analysis

All data were expressed as mean ± standard error. The groups were compared by one-way ANOVA analysis followed by Tukey's post-test. SPSS 22.0 software (SPSS, Chicago, IL, USA) was used for all analyses, and *P* < 0.05 was considered statistically significant.

## Results

### Preparation and characterization of iELPs

**Figure 1:**
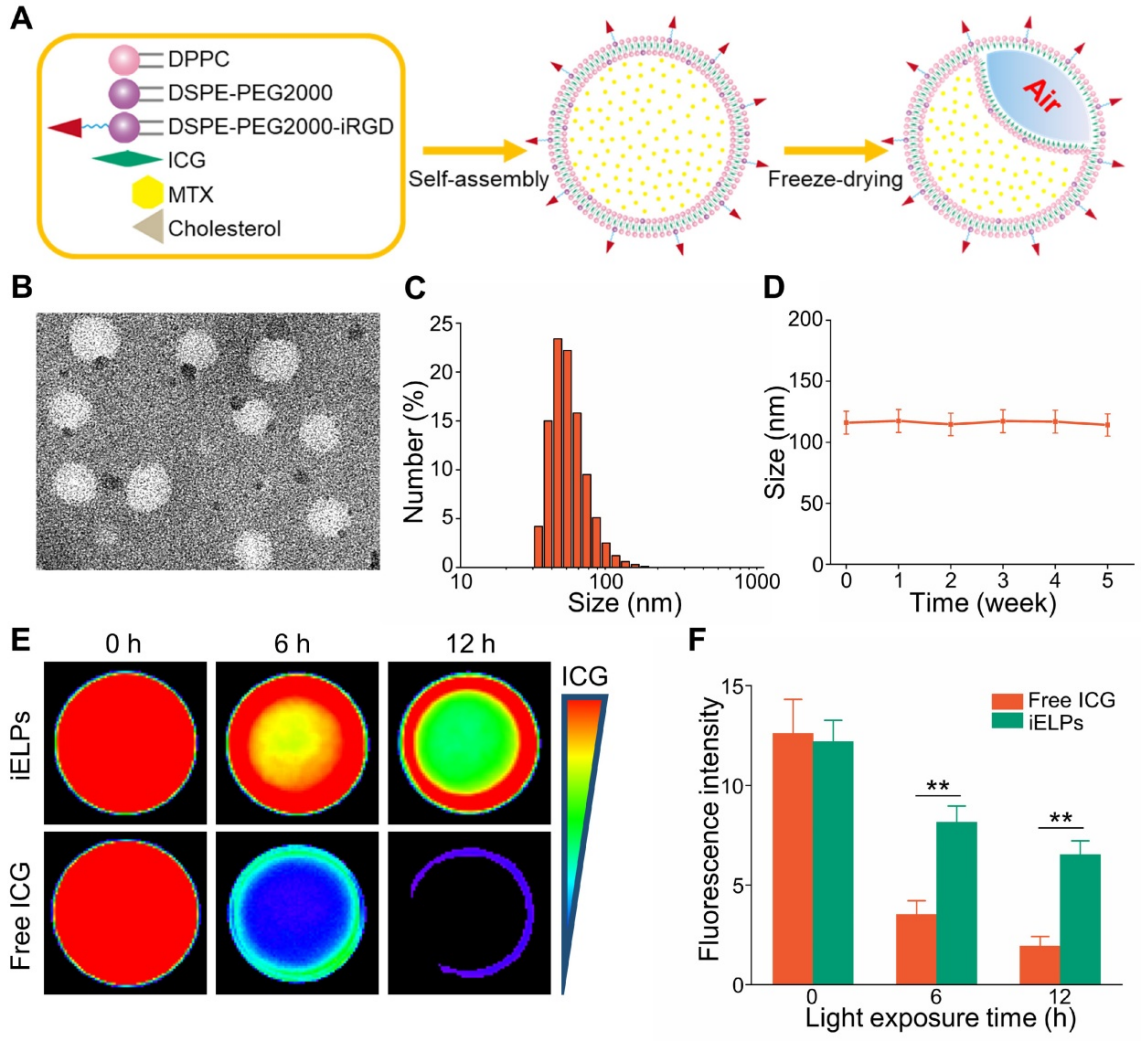
** Preparation and characterization of iELPs.** (**A**) Schematics of iELP preparation. (**B**) TEM micrographs showing iELP morphology. (**C**) Size distribution of iELPs. (**D**) Changes in the mean diameter of iELPs following storage at 4°C for varying durations. (**E**) Fluorescence images of iELPs and free ICG after light exposure and (**F**) quantification (**P < 0.01).

The iELPs was successfully prepared by thin-film hydration and freeze drying (Figure 1A), and exhibited a well-defined spherical shape and uniform distribution (Figure 1B). The average diameter, zeta potential and size distribution of the iELPs were 113.35 ± 4.61 nm, -10.07 ± 4.28 mv and 0.22 ± 0.01, respectively (Figure 1C). The encapsulation efficiencies for ICG and MTX were 68.72% ± 0.64% and 99.14% ± 0.82%, respectively (Table 1). The characterization of ELPs is summarized in Table 1. In addition, no significant difference was observed in the mean diameter of the liposomes after 5 weeks of storage at 4°C (Figure 1D), indicating good stability. Moreover, the stability of iELPs in H_2_O, phosphate buffer solution (PBS) and fetal bovine serum (FBS) was evaluated by drug release behaviors. As shown in figure S1, the drug accumulative leakage was lower than 15% in PBS and H_2_O, and below 30% after 6 h incubation in FBS, suggesting a certain stability of iELPs in blood circulation. The fluorescence intensity of the iELPs was also 1.93-fold higher than that of free ICG at room temperature over a period of 12 hours (Figure 1E & F,* P* < 0.05), indicating that iELPs stabilize and enhance ICG fluorescence by preventing oxidation 18.

### The iELPs release MTX in a controlled ultrasound-triggered manner

**Figure 2:**
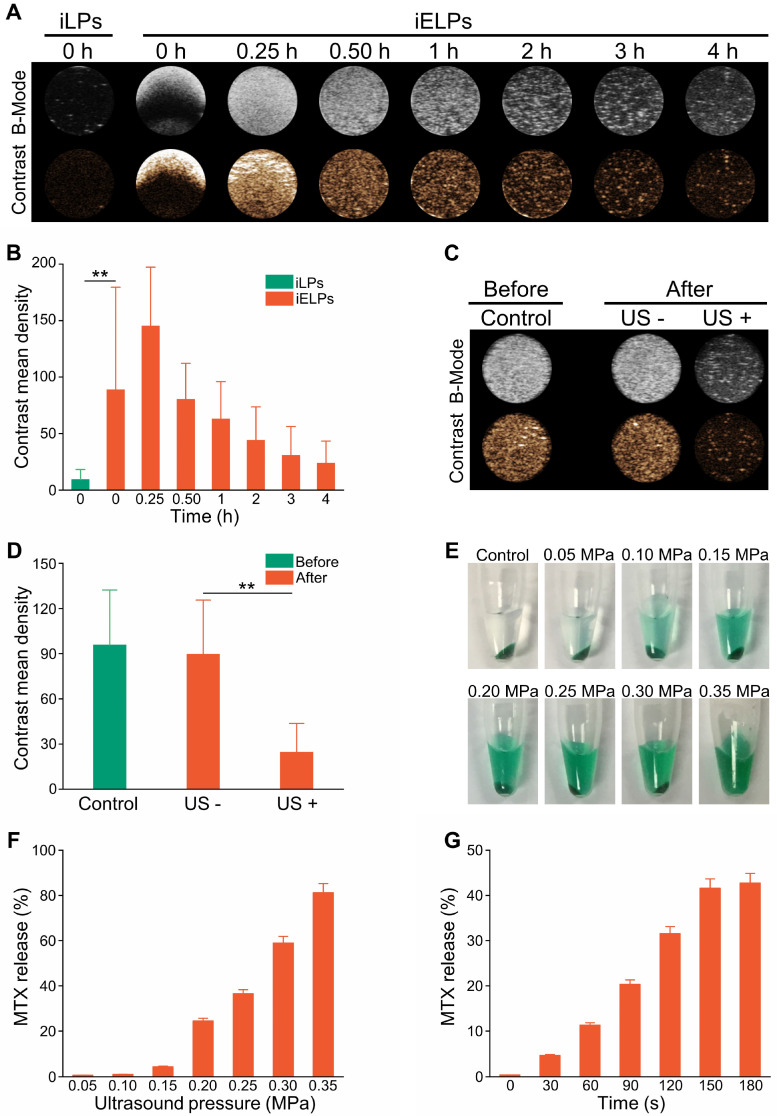
** Ultrasonic characterization of iELPs.** (**A**) *In vitro* B-mode and contrast ultrasound images of the agar phantom embedded with iELPs for varying durations without sonication. (**B**) Gray intensities of the iLPs and iELPs in the absence of sonication (**P < 0.01). (**C**)* In vitro* B-mode and contrast ultrasound images of the agar phantom embedded with iELPs before and after sonication. (**D**) Gray intensities of iELPs before and after sonication (**P < 0.01). (**E-G**) Amount of MTX released from the iELPs when sonicated at different acoustic pressures (E, F) and for varying durations (G).

Ultrasound imaging showed high contrast images of iELPs but not of the control of non-lyophilized targeted liposomes (iLPs) over a period of 4 h in the absence of sonication (Figure 2A, B; *P* < 0.05). However, low-frequency ultrasound significantly weakened the contrast images of iELPs (Figure 2C & D; *P* < 0.05), indicating that the liposomes were likely disrupted by sonication. Consistent with this, MTX was released steadily from the sonicated iELPs (Figure 2E), and the release rate increased from 0.62% ± 0.03% to 81.23% ± 4.06% depending on the acoustic pressure (Figure 2F) and time (Figure 2G). In contrast, no MTX was released from the non-sonicated liposomes. Furthermore, an insignificant amount of MTX was leaked from the iELPs after 24 h of dialysis in the absence of ultrasound irradiation (Figure S1). Taken together, MTX was stably encapsulated in the iELPs and could only be released following ultrasonic stimulus. This bodes well for *in vivo* applications since the drug would not leak prematurely and be released in a controlled manner by adjusting the sonication pressure and duration.

### The iELPs specifically target HUVECs

**Figure 3:**
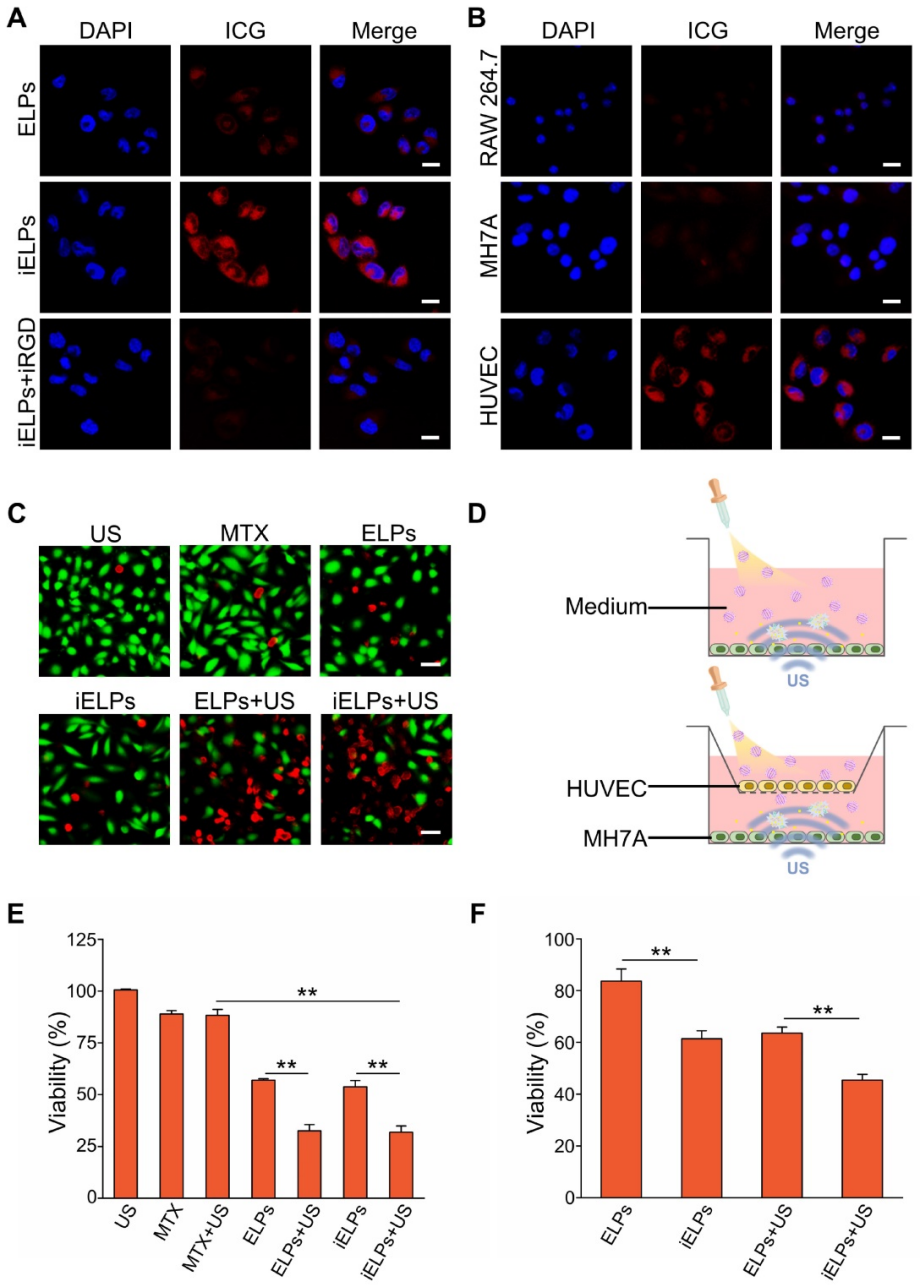
** Targeted cellular uptake and cytotoxicity of iELPs.** (**A**) Confocal fluorescence images in HUVECs showing cellular uptake of ICG after incubation with ELPs, iELPs or iRGD+iELPs. Blue - DAPI and red - ICG. (Scale bar = 20 µm.) (**B**) Confocal fluorescence images showing cellular uptake of iELPs in different cell types. (Scale bar = 20 µm.) (**C**) Fluorescence images of differentially-treated MH7A. Viable cells are stained green with calcein-AM, and dead cells are stained red with PI. (Scale bar = 50 µm.) (**D**) Schematic illustration of assaying *in vitro* toxic to MH7A. (**E, F**) Percentage of viable MH7A cells after different treatments (**P < 0.01).

As shown in Figure S2, HUVECs showed high expression of integrin αvβ3 compared to the relatively lower levels in other cells. HUVECs incubated with iELPs showed significantly stronger fluorescence signals compared to those exposed to ELPs, indicating greater ICG uptake in the former. Furthermore, pre-blocking the HUVECs with free iRGD markedly decreased the fluorescence intensity of the iELPs (Figure 3A). This clearly indicated that the HUVECs absorbed the iELPs through the specific binding between iRGD and the αvβ3 integrin, and pre-incubation with excess iRGD significantly decreased the number of available receptors for iELPs. To further establish the cellular specificity of the iELPs, they were incubated with HUVECs, the RAW 264.7 macrophages and the RA synoviocyte line MH7A. The HUVECs showed the strongest fluorescence, followed by MH7A and the macrophages which fluoresced weakly (Figure 3B). Also, the results of flow cytometer demonstrated that the iELPs in HUVECs group exhibited the highest uptake efficiency and fluorescence intensity than other drug or cell groups, which further confirmed the specific binding ability of iELPs to HUVECs (Figure S3). Taken together, the iELPs can specifically target HUVECs via the iRGD peptide.

### The iELPs are toxic to RA syniocytes upon sonication

The effects of sonication time and acoustic pressure on the MH7A cells were first standardized. As shown in Figure S4A, the viability of MH7A cells decreased from 95.43% ± 4.77% to 15.13% ± 0.81% as the acoustic pressure increased from 0.20 MPa to 0.30 MPa for 90 s duration, while < 0.20 MPa had no effect on cell survival. At the constant acoustic pressure of 0.20 MPa, the percentage of viable MH7A cells decreased from 95.41% ± 4.77% to 86.91% ± 4.34% (Figure S4B) as the sonication time was increased from 150 s to 180 s, while durations shorter than 150 s had no significant effect. Accordingly, we selected the sonication parameters of 0.20 MPa and 90 s for subsequent *in vitro* experiments. As shown in the calcine AM/PI-stained fluorescence images in Figure 3C, the sonicated groups had notably more dead cells compared to others. Without donor wells implanted with HUVECs, the percentage of viable MH7A cells treated with iELPs decreased from 53.78% ± 3.01% in the absence of sonication to 31.84% ± 3.02% after sonication. Similarly, sonication also decreased the viability of the ELPs-treated cells from 58.62% ± 0.74% to 34.47% ± 2.86% (*P* < 0.05 for both). Furthermore, the survival rates were similar between the iELPs and ELPs-treated cells regardless of sonication (*P* > 0.05; Figure 3D & E). However, viability of MH7A cells significantly reduced between the iELPs and ELPs-treated cells, with donor wells implanted with HUVECs (*P* < 0.05; Figure 3D & F). Taken together, ultrasound-triggered release of MTX significantly improved the therapeutic efficiency of iELPs and ELPs in MH7A cells, which was not influenced by the iRGD peptide. The difference in MH7A cellular uptake of MTX is attributed to HUVECs targeting capacity of iELPs.

### The iELPs accumulate selectively in the arthritic lesions and enable NIR fluorescence imaging

**Figure 4:**
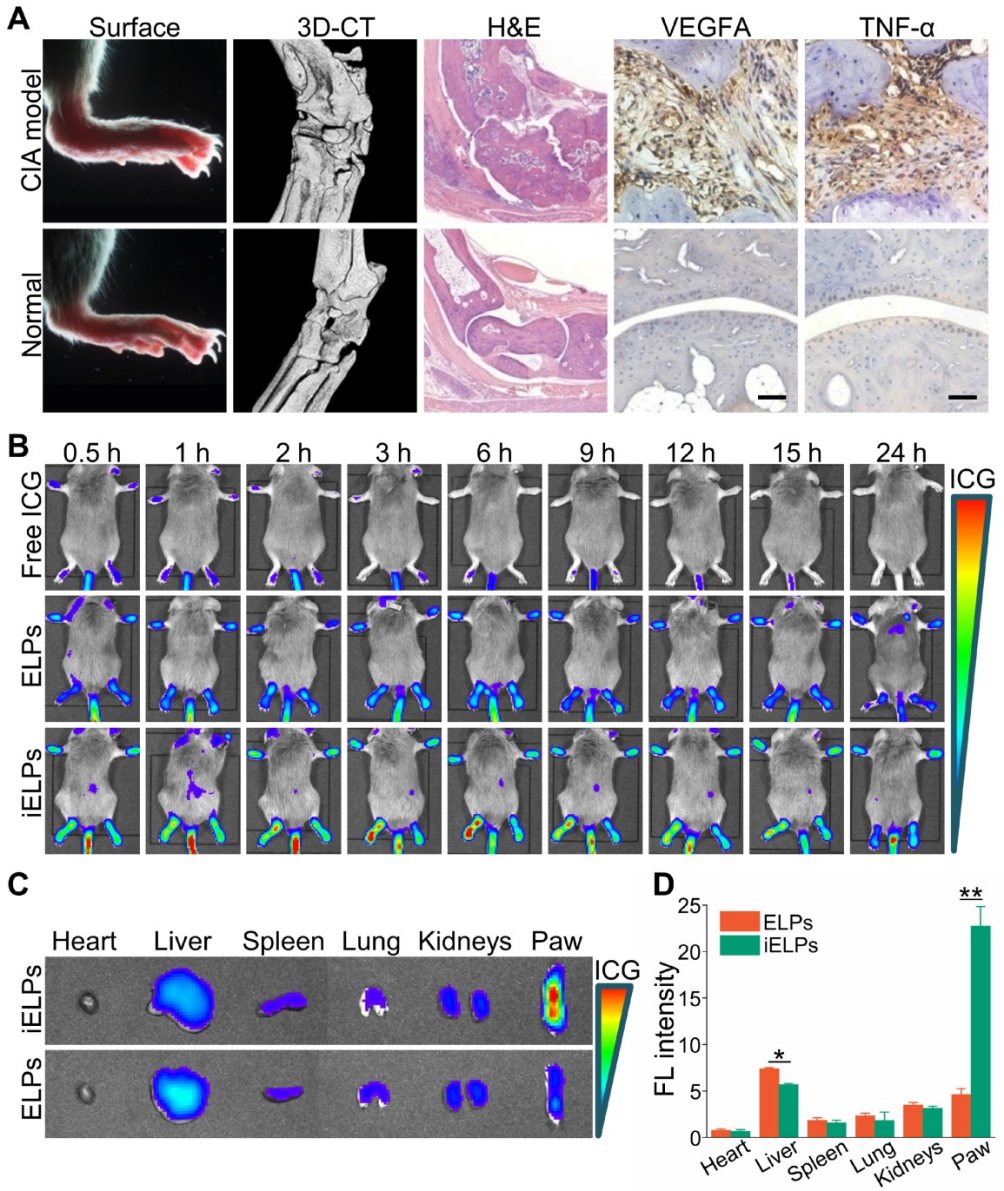
** Evaluation of *in vivo* RA model and NIR imaging by the liposomes.** (**A**) Representative images the paws, ankle micro-CT 3D reconstruction, the H&E and immuno-stained articular sections of normal and CIA model mice. (Scale bar = 100 µm.) (**B**) NIR images of CIA mice at different time points after injection with free ICG, ELPs or iELPs. (**C**) Biodistribution of iELPs and ELPs in the main organs at 24 h after injection, and (**D**) comparison of fluorescence intensities in the paws (**P < 0.01). Abbreviations: FL: fluorescence.

The CIA model was successfully established, with mice showing destructive polyarthritis, redness and swelling of paws and invasive synovitis. Furthermore, VEGFA and TNF-α were over-expressed in the arthritic tissues (Figure 4A).

The mice and porcine tissue pieces subjected to low-frequency sonication at < 0.25 MPa for 3 minutes showed no obvious swelling in the paws or any other damage in healthy tissues. However, acoustic pressures greater than 0.30 MPa resulted in severe swelling, ulceration and even gangrene in the limbs (Figure S5 & S6). Therefore, 0.20 MPa was selected for the subsequent experiments.

**Figure 5:**
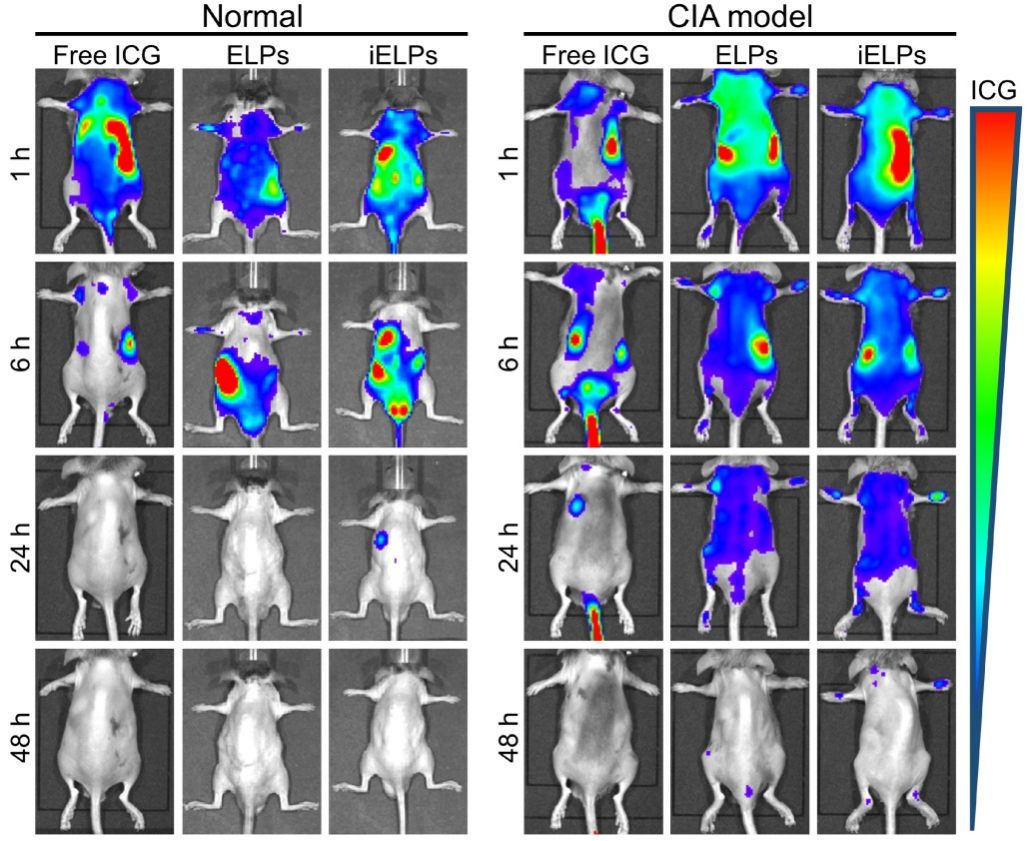
* In vivo* distribution of different drugs. *in vivo* changes of distribution between normal mice and CIA model mice after injection of free ICG, ELPs or iELPs.

The mice with clinical arthritic index of 10 to 12 were injected with free ICG, ELPs or iELPs, and imaged by NIR light at varying time points after injection. The fluorescence signal of ICG was detected in the inflamed paws within 30 minutes of injection in all groups. However, the fluorescence intensity in the iELPs group was respectively 2.64-fold and 4.21-fold higher than that in the ELPs and free ICG groups. In addition, the peak fluorescence signals in all groups were observed after 6 h, and were 2.72-fold and 7.87-fold higher in the iELPs group compared to ELPs and free ICG groups respectively. The iELPs maintained the higher fluorescence intensity throughout the 24 h period (Figure 4B & S7). Therefore, 6 h after rejection was selected as the sonication duration. Strong fluorescent signals were also detected in the liver, paws and kidneys. Furthermore, the fluorescence in the inflamed paws of the iELPs-treated mice was 7.72-fold higher while that in the liver was 0.77-foldlower compared to that in the ELPs group. No significant differences were seen with the other organs (Figure 4C & D). For normal mice, the fluorescence signal did not show a great change in paws, and all of three drug metabolisms were faster than CIA model mice. The liposome-encapsulated ICG showed longer retention *in vivo* compared to the free drug (Figure 5). After sonication, the fluorescence intensity in the iELPs+US group was 3.22-fold higher than that in the iELPs group (Figure S8). Taken together, the presence of ICG in the liposomes enabled NIR fluorescence imaging, the iELPs were released following ultrasonic stimulus and the iRGD peptide improved targeted drug distribution *in vivo*.

### The iELPs show high therapeutic efficacy in the RA model upon ultrasonic stimulation

**Figure 6:**
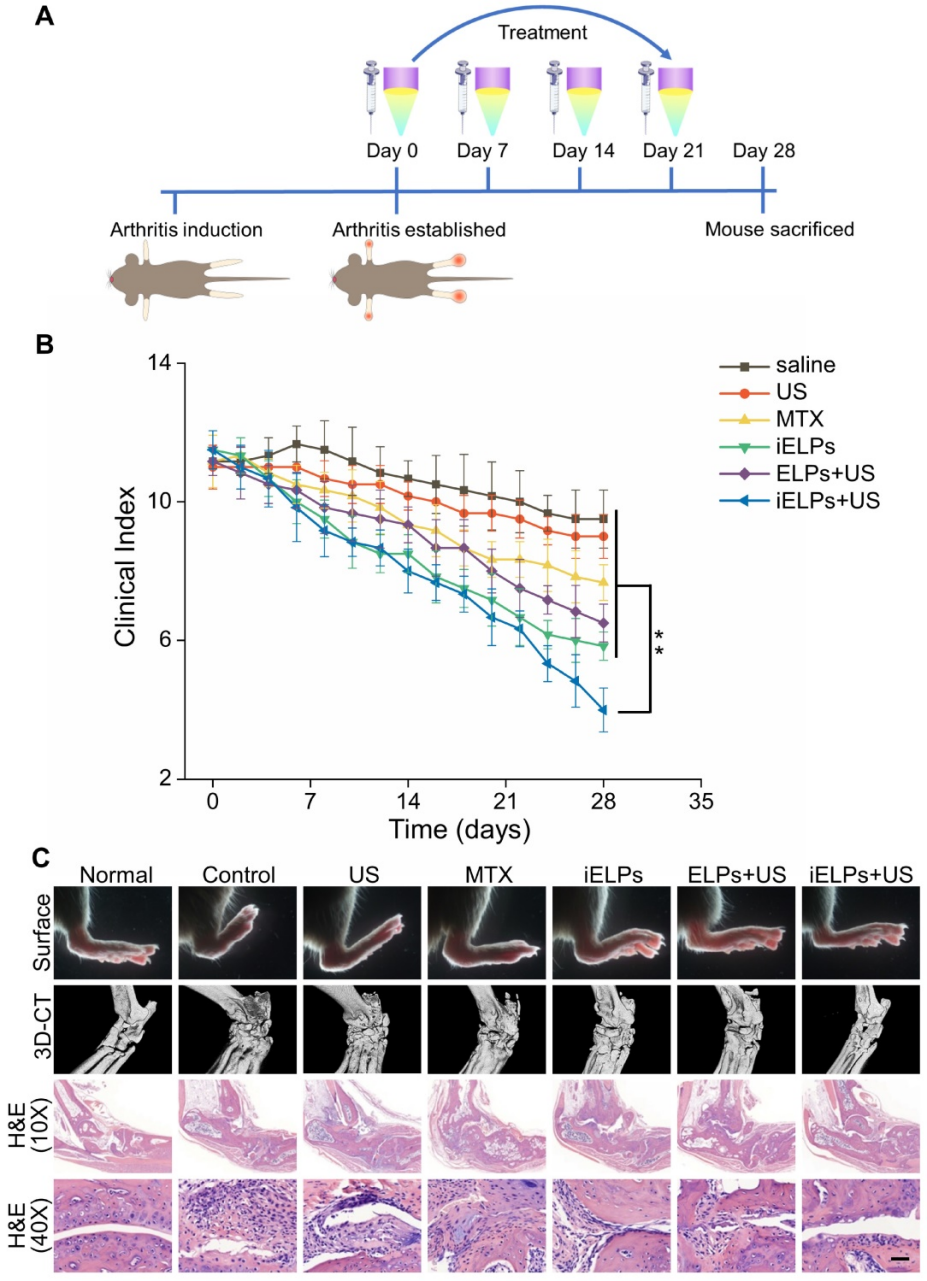
** Evaluation of *in vivo* therapeutic efficacy and biosafety of iELPs.** (**A**) Schematics of arthritis induction and therapeutic regimen. (**B**) Time-dependent change in arthritic clinical index in the different treatment groups (**P < 0.01). (**C**) Macroscopic, micro-CT 3D reconstruction and histological images of joint tissues of normal and CIA mice after 28 days of the different treatments. (Scale bar = 100 µm).

The CIA mice were subjected to different treatments, and the groups and schedule are shown in Figure 6A. The mean clinical index was similar across the groups prior to treatment (*P* > 0.05), and showed the greatest decrease in mice treated with the combination of iELPs and sonication (G6). A significant improvement was also seen in mice treated with iELPs without sonication (G4), or with ELPs and sonication (G5). The lower post-treatment average clinical index in G6 compared to G4 indicated that sonication-induced controlled drug release from the liposomes improved therapeutic efficacy. Furthermore, G6 had significantly lower clinical index compared to G5, indicating that iRGD-mediated homing of the liposomes to the target sites also augmented drug effect. The clinical arthritic indices decreased in these groups in a time-dependent manner (Figure 6B). Injection of free MTX (G3) had only a minor therapeutic effect, indicating that its efficacy was significantly improved by liposomal encapsulation which increased targeted accumulation. Finally, low-frequency ultrasound alone (G2) had no significant effect on the arthritic joints of RA mice (G2). The data are summarized in Table 2.

Macroscopic examination of the ankle joint of normal healthy mice indicated natural shape, no obvious local redness or swelling, ruddy epidermis, good blood flow, and good flexibility. In contrast, the ankle joints of the G1, G2 and G3 mice were stiff, inflamed, pale and deformed. The G4 and G5 mice showed slight improvement with a small range of motion, and only localized redness and swelling. The ankle joint of G6 mice showed maximum improvement, with normal shape, good flexibility and punctate redness in the toe tips, although the blood flow was still sub-optimal (Figure 6C; upper panel). Consistent with the visual examination, the Micro-CT 3D reconstruction images of the normal mouse ankle showed clear structure, smooth cortex and uniform joint space. In the G1, G2 and G3 mice, the ankle joints were disordered, the joint space had disappeared, and severe cortical erosion and bone hyperplasia were seen. In the G4 and G5 mice also, the morphology of the ankle joints was not regular and the local joint space was absent. However, cortical erosion was limited to the toes. The ankle joints in the G6 mice were significantly clearer and except the ends of the articular cavity, the cortex was smooth (Figure 6C). Histopathological examination showed no obvious signs of inflammation or cartilage destruction in the ankle joints of normal mice. The articular tissues of the G1, G2 and G3 mice showed synovial hyperplasia in and around the joint space, along with extensive angiogenesis and severe destruction of cartilage and bone cortex. The G4 and G5 mice also showed varying degrees of synovial hyperplasia, neovascularization, local cartilage and bone cortex destruction. Inflammatory cell infiltration and angiogenesis decreased significantly in the ankle joint space of the G6 mice, with no obvious growth or destruction of the cartilage and bone tissue (Figure 6C). Finally, the heart, liver, spleen, lung and kidney tissues showed regular arrangement and absence of necrotic lesions in any of the groups, indicating that neither therapeutic modality was toxic (Figure S9).

## Discussion

This study constructed echogenic MTX-loaded liposomes that showed targeted accumulation, NIR fluorescence imaging and controlled ultrasound-triggered drug release, which translated to significant therapeutic effects in an *in vivo* RA model. Low-frequency ultrasound leads to a cavitation effect that not only disrupts the liposomes and release the drug, but also weakens the tight junctions of the endothelium. Studies showed that ultrasound increases potassium influx into endothelial cells through ion channels, which depolarizes the membrane and opens the voltage-dependent calcium channels, leading to calcium influx and activation of endothelial NO synthase. The subsequent increase in NO levels in the endothelial cells opens the tight junctions and increases vascular permeability, which allows the extravasation of nanoparticles 19. In our model therefore, the iELPs that accumulated in the vascular endothelium released the encapsulated MTX in the surrounding inflammatory tissues, thus enhancing local drug concentration to therapeutically effective levels.

Ultrasound is a mechanical wave that can enhance blood perfusion in the surrounding tissues and accelerate targeted drug delivery independent of its thermal effects. In fact, Belcik et al. showed that ultrasound alone can increase intramuscular perfusion by 2-fold 20. In addition, the microfluidic effects of ultrasound exert shear forces on vascular ECs and upregulate NO synthase, resulting in vasodilation as described above. However, the RA mice in our study that received only ultrasound showed no improvement compared to saline control. Thus, the primary function of ultrasound in this model was to promote drug penetration. In our previous study on a breast cancer model as well, we found ultrasonic irradiation significantly increased the accumulation of paclitaxel-loaded microbubbles in the tumor tissues compared to the non-irradiated group 21.

Controlled drug release is a crucial factor that determines the efficacy of targeted therapy, and depends on the optimum stimulus and nanoparticle design. The iELPs had air-filled cavities with phospholipid bilayers that were destroyed by low frequency sonication, indicating that the encapsulated drugs can be released from these liposomes in a controlled manner by adjusting the acoustic parameters. Optimal retention and enrichment of nanoparticles at the lesions is also essential for their therapeutic success. Similar to the enhanced permeability and retention effect (EPR) effect of solid tumors, nanoparticles exudate from hyperosmotic blood vessels and selectively gather in periarticular inflammatory tissue, the phenomenon known as inflammatory cell isolation after vascular system exudation (extravasation across leaky vasculature followed by sequestration by inflammatory cells, ELVIS) 22-24. The nanoparticles in proper size can be passively extruded from permeable blood vessels into adjacent tissues, and are mainly taken up by macrophages and fibroblasts after entering the joint cavity through the leaky vasculature. During RA progression, the inflammatory cells and factors stimulate neo-angiogenesis and enhance the permeability of the vascular endothelium 25, 26, which allows molecules smaller than 500 nm to pass through the intercellular gaps and enter the inflamed tissues 27. However, nanoparticles smaller than 10 nm or larger than 200 nm are rapidly cleared by the kidney or spleen 28. Therefore, nanoparticles with diameter between 100 and 300 nm are preferred for therapeutic applications 29. The average diameter of the iELPs and ELPs was 100 nm, which increased their accumulation in the periarticular inflammatory tissue. In addition, fluorescence imaging showed that while the free MTX was cleared within 24 h of injection, the iELPs and the ELPs were retained for longer periods. The drug did not accumulate in the paws of normal mice, and was rapidly cleared compared to that in the CIA model mice, further confirming the ELVIS effect in inflamed tissues.

The third requirement for effective targeted therapy is tissue/cell specificity of the nanoparticles. The mean fluorescence intensity of the iELPs in the RA joint was significantly higher than that of the ELPs, indicating that the iRGD peptide in the former can effectively target the liposomes to the inflamed endothelium by binding to the αvβ3 integrin. Lee et al. also observed greater affinity of RGD-loaded Au nanoparticles to arthritic lesions 12. iRGD, one of RGD members, binds to the integrin ανβ3 receptor is followed by disintegration of the latter, which improves cellular penetration of the drugs and therefore improves its therapeutic activity 30, 31. Thus, the presence of both the angiogenesis-specific recruitment element (RGD) and the recognition sequence (CRGDK) distinguishes iRGD from some previously described RGD peptides.

Bio-imaging is increasingly becoming indispensable for disease diagnosis and surveillance 32. Compared to X-rays, NIR fluorescence imaging is convenient, sensitive and rapid, and therefore more suitable for real time tracking and image-guided therapy. Live imaging of the differentially-treated RA mice showed that the iELPs accumulated at the arthritic lesions where they were retained for more than 6 hours. Although fluorescence imaging is a reliable and convenient method for monitoring nanoparticle distribution *in vivo*, it has a short time window which may reduce the accuracy of image-guided therapy 33. Finally, the iELPs were also biocompatible and showed no systemic toxicity.

Despite the encouraging results shown by the iELPs, there are several limitations in this study that ought to be addressed. Firstly, the CIA model does not accurately simulate human RA since it lacks the immune-pathological factors like antinuclear antibody (ANA), C-reactive protein (CRP) and rheumatoid factor (RF) associated with RA. In addition, the encapsulation of ICG in the liposome is not completely leak proof, and thus may not precisely track liposome movement *in vivo*.

## Conclusion

This study constructed acoustic iRGD-modified MTX-loaded liposomes that targeted the inflammatory joints in RA mice, released the drug in a controlled manner upon sonication, and enabled NIR fluorescence imaging. The iRGD peptide conjugation significantly enhanced the accumulation and enrichment of iELPs in arthritic joints. This novel nanoplatform can maximize the therapeutic efficacy of MTX against RA and minimize systemic side effects in a minimally invasive manner, and should be explored further for the diagnosis and targeted therapy of other chronic diseases.

## Supplementary Material

Supplementary figures and tables.

## Figures and Tables

**Scheme 1:**
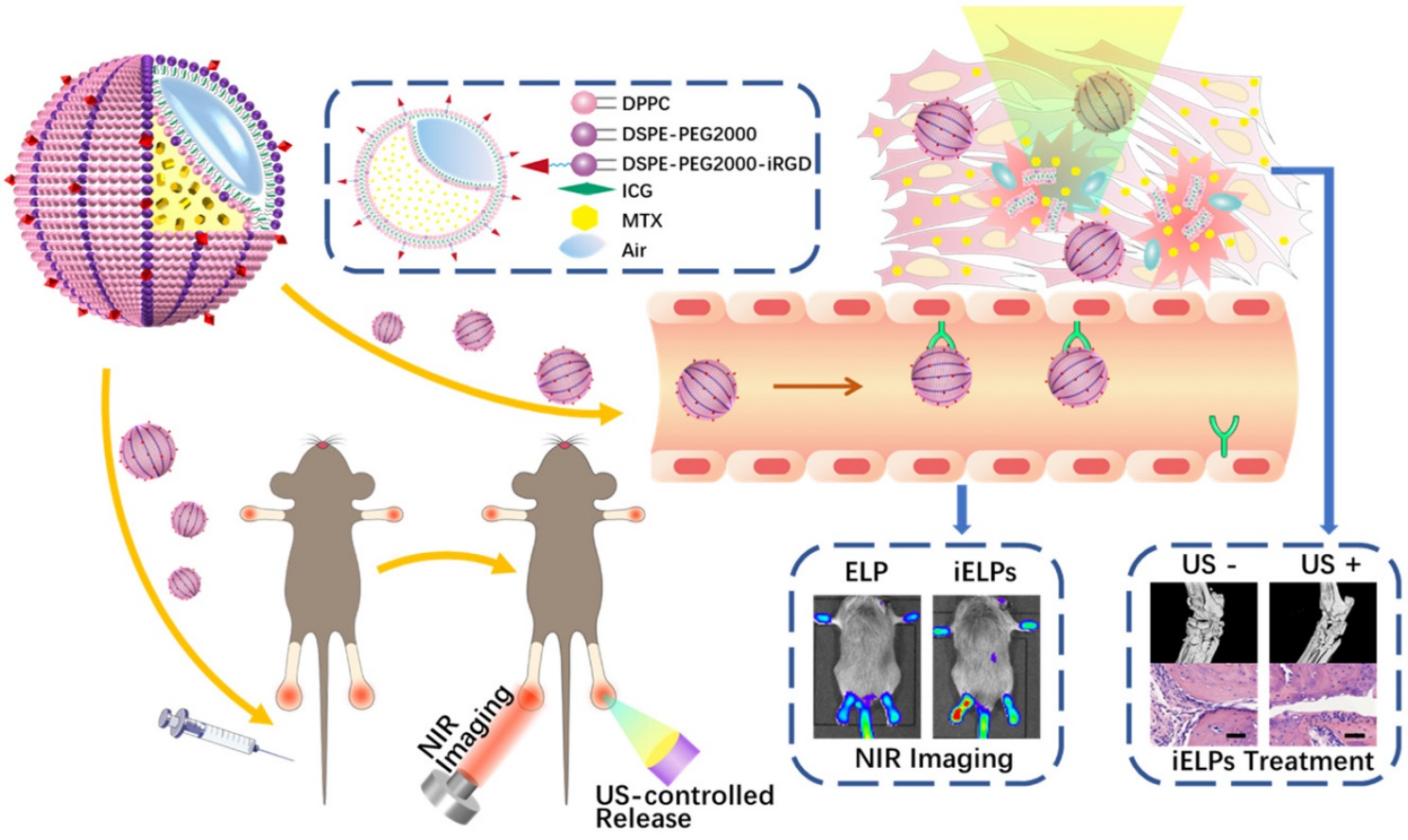
The schematic illustration of iELPs and the mechanism of NIR fluorescence imaging and treatment.

**Table 1:** General properties of iELPs and ELPs

Liposomes	Diameter (nm)	Zeta potential (mv)	Polydispersity (PDI)	MTX EE (%)	ICG EE (%)
ELPs	98.32 ± 0.72	-11.23 ± 8.92	0.15 ± 0.01	67.64 ± 0.72	98.30 ± 0.56
iELPs	113.35 ± 4.61	-10.07 ± 4.28	0.22 ± 0.01	68.72 ± 0.64	99.14 ± 0.82

**Table 2:** Comparison of the different treatment regimens in CIA mice

Group (n=6)	Injected content^a^	Ultrasonic irradiation^b^	Clinical index before treatment	Clinical index after treatment
G1	Saline	—	11.17 ± 0.41	9.50 ± 0.84
G2	—	+	11.00 ± 0.63	9.00 ± 0.63
G3	MTX solution	—	11.17 ± 0.75	7.67 ± 0.52
G4	iELPs	—	11.50 ± 0.55	5.83 ± 0.41
G5	ELPs	+	11.17 ± 0.41	6.50 ± 0.55
G6	iELPs	+	11.50 ± 0.55	4.00 ± 0.63

*a,* The liposomes/MTX were administered in a volume of 0.1 ml by tail vein injection; *b,* The arthritic joint was exposed to ultrasonic irradiation for 3 min at 6 h post-injection, and all treatments were repeated 7 days later for 4 times.

## Abbreviations

ANA: antinuclear antibody
CCK-8: Cell Counting Kit-8
CIA: the collagen-induced arthritis
CRP: C-reactive protein
DAPI: '4, 6-diamidino-2-phenylindole
ECs: endothelial cells
EE: encapsulation efficiency
ELPs: the echogenic liposomes containing MTX and ICG decorated
ELVIS: extravasation across leaky vasculature followed by sequestration by inflammatory cells
EPR: enhanced permeability and retention effect
FBS: fetal bovine serum
FL: fluorescence
H&E: hematoxylin-eosin staining
HUVEC: human umbilical vein endothelial cell line
ICG: indocyanine green
iELPs: the echogenic liposomes containing MTX and ICG decorated with iRGD peptide
iLPs: the control of non-lyophilized liposomes containing MTX and ICG decorated with iRGD peptide
LPs: the control of non-lyophilized liposomes containing MTX and ICG decorated
Micro-CT: micro-computed tomography
MTX: Methotrexate
NIR: near-infrared
NO: nitric oxide
PBS: phosphate buffer saline
PI: propidium iodide
RA: rheumatoid arthritis
RF: rheumatoid factor
TEM: transmission electron microscopy
TNF-α: tumor necrosis factor alpha
US: ultrasound
VEGFA: vascular endothelial growth factor-A
