# Supplementary material for: Near-infrared fluorescence imaging-guided focused ultrasound-mediated therapy against Rheumatoid Arthritis by MTX-ICG-loaded iRGD-modified echogenic liposomes
Source: Theranostics. 2020 Aug 8;10(22):10092–105. doi: 10.7150/thno.44865 (PMC7481417; doi:10.7150/thno.44865)
Supplement: Supplementary file 1 — Supplementary figures and tables. [file thnov10p10092s1.pdf]

Supporting Information

Near-infrared fluorescence imaging-guided focused ultrasound-mediated therapy against rheumatoid arthritis by MTX-ICG-loaded iRGD-modified echogenic liposomes

Haohan Wu[1,2,†], Yanni He[1,2,†], Hao Wu[2], Meijun Zhou[2], Zhili Xu[2], Ran Xiong[2], Fei Yan[2,3,*], Hongmei Liu[2,1,*]

[1] The Second School of Clinical Medicine, Southern Medical University, Guangzhou 510515, China

[2] Department of Ultrasound, Institute of Ultrasound in Musculoskeletal Sports Medicine, Guangdong Second Provincial General Hospital, Guangzhou 510317, China

[3] CAS Key Laboratory of Quantitative Engineering Biology, Shenzhen Institute of Synthetic Biology, Shenzhen Institutes of Advanced Technology, Chinese Academy of Sciences, Shenzhen 518055, China

[†] These two authors equally contributed to this work.

[*] Correspondence authors:

Prof. Hongmei Liu: Tel./fax 86-19924270557 and E-mail lmeihong@fimmu.com;

Prof. Fei Yan: Tel./fax 86-755-86392284 and E-mail fei.yan@siat.ac.cn.

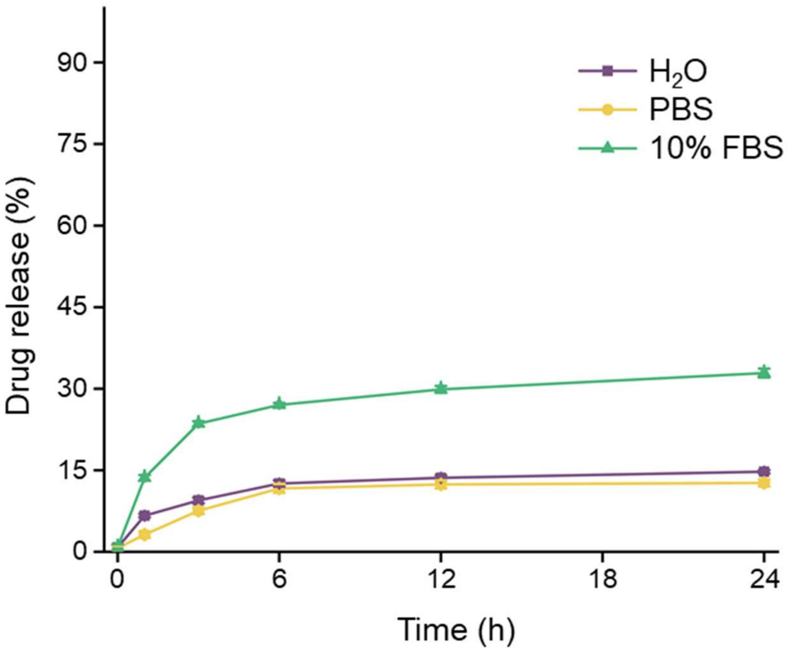

Figure S1. Drug release profiles of iELPs in H_2O, PBS and 10% FBS after 24 h incubation.

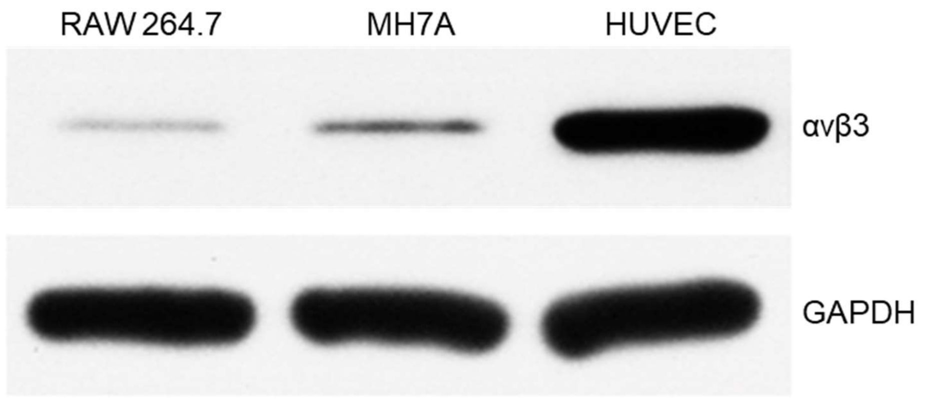

Figure S2. The assessment of integrin αvβ3 expression for three cells through Western blotting.

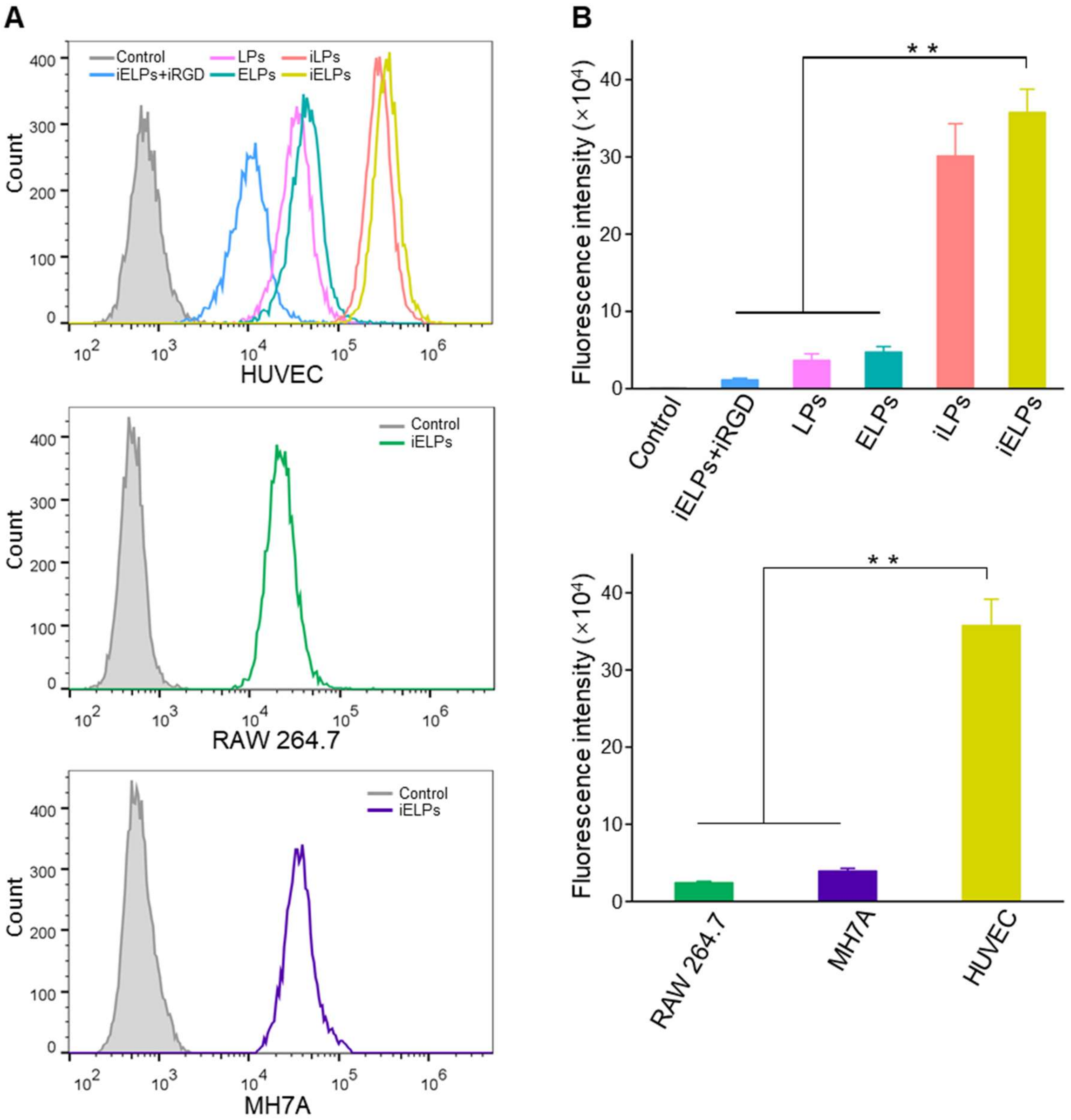

Figure S3. (A) The uptake analysis of HUVECs, RAW 264.7 cells and MH7A cells by flow cytometry. (B) Fluorescence intensity of HUVECs, RAW 264.7 cells and MH7A cells from flow cytometric analysis (***P* < 0.01).

Abbreviations: iLPs: the control of non-lyophilized liposomes containing MTX and ICG decorated with iRGD peptide; LPs: the control of non-lyophilized liposomes containing MTX and ICG decorated.

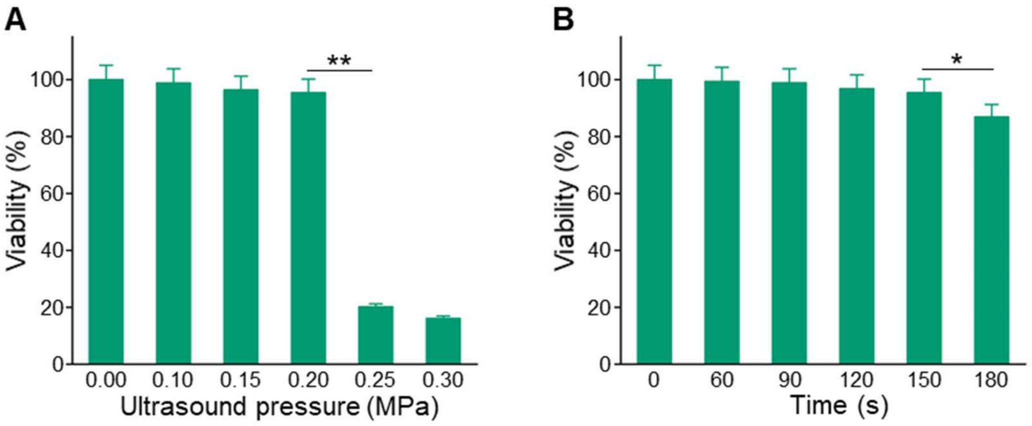

Figure S4. (A, B) *In vitro* cytotoxicity of low frequency ultrasound at varying acoustic pressure (A) and duration (B) (*$P < 0.05$, **$P < 0.01$).

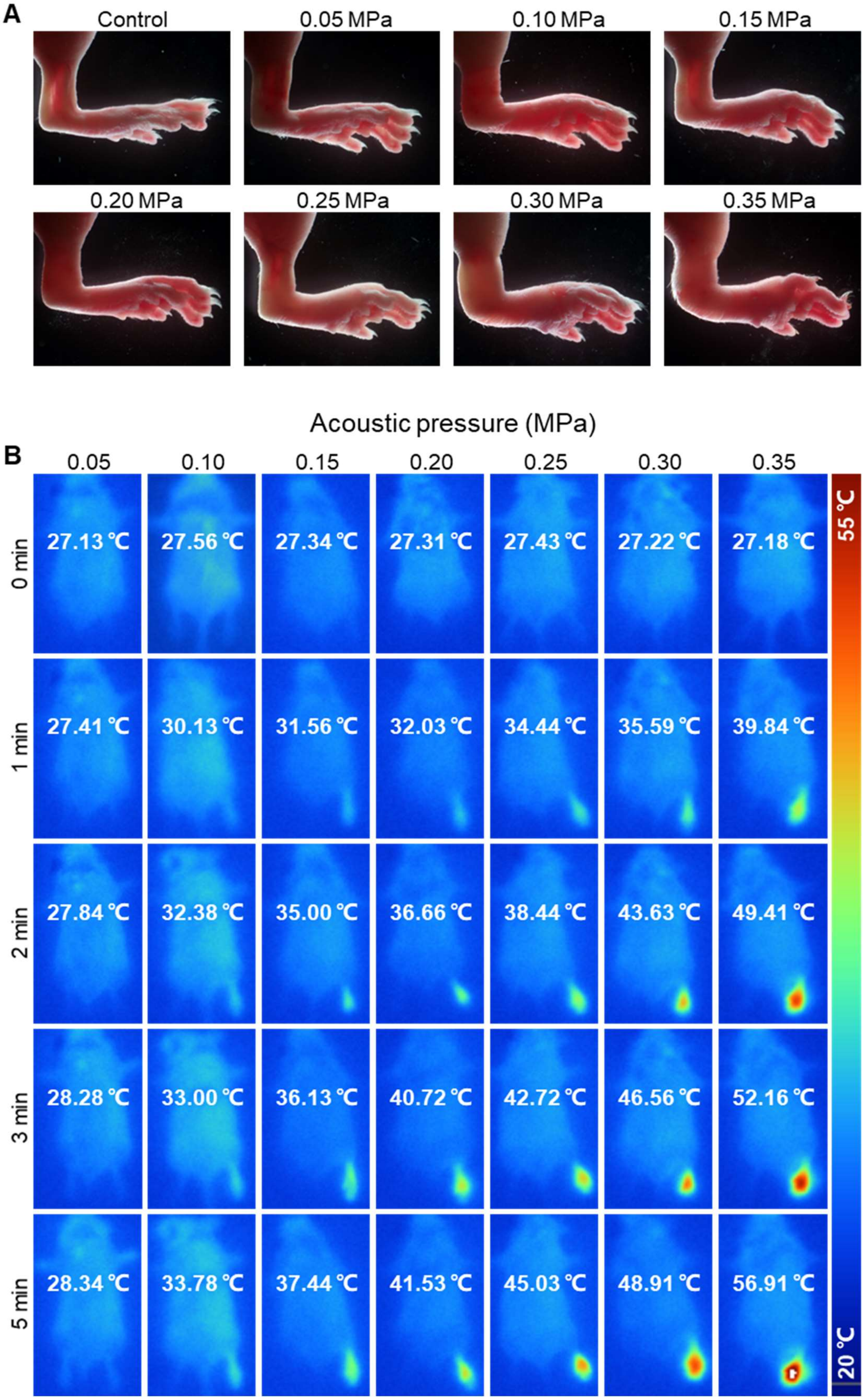
A
Control
0.05 MPa
0.10 MPa
0.15 MPa
0.20 MPa
0.25 MPa
0.30 MPa
0.35 MPa
B
Acoustic pressure (MPa)
0.05
0.10
0.15
0.20
0.25
0.30
0.35
0 min
27.13 ℃
27.56 ℃
27.34 ℃
27.31 ℃
27.43 ℃
27.22 ℃
27.18 ℃
1 min
27.41 ℃
30.13 ℃
31.56 ℃
32.03 ℃
34.44 ℃
35.59 ℃
39.84 ℃
2 min
27.84 ℃
32.38 ℃
35.00 ℃
36.66 ℃
38.44 ℃
43.63 ℃
49.41 ℃
3 min
28.28 ℃
33.00 ℃
36.13 ℃
40.72 ℃
42.72 ℃
46.56 ℃
52.16 ℃
5 min
28.34 ℃
33.78 ℃
37.44 ℃
41.53 ℃
45.03 ℃
48.91 ℃
56.91 ℃
55 ℃
20 ℃

Figure S5. (A) Changes in the articular surface after sonication at different acoustic pressures. (B) Realtime photothermal images of acoustic mice.

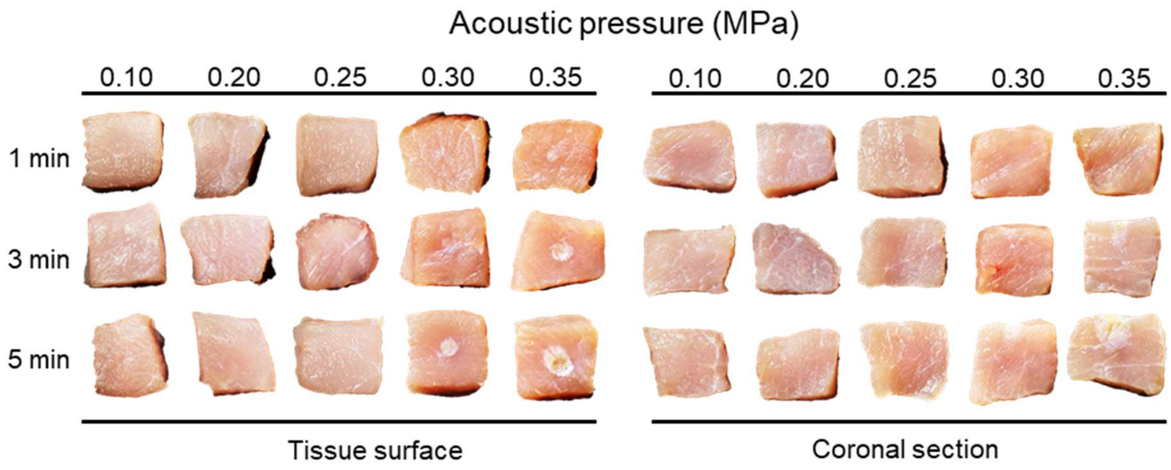

Figure S6. Porcine tissue damage and temperature changes varying acoustic pressure and duration.

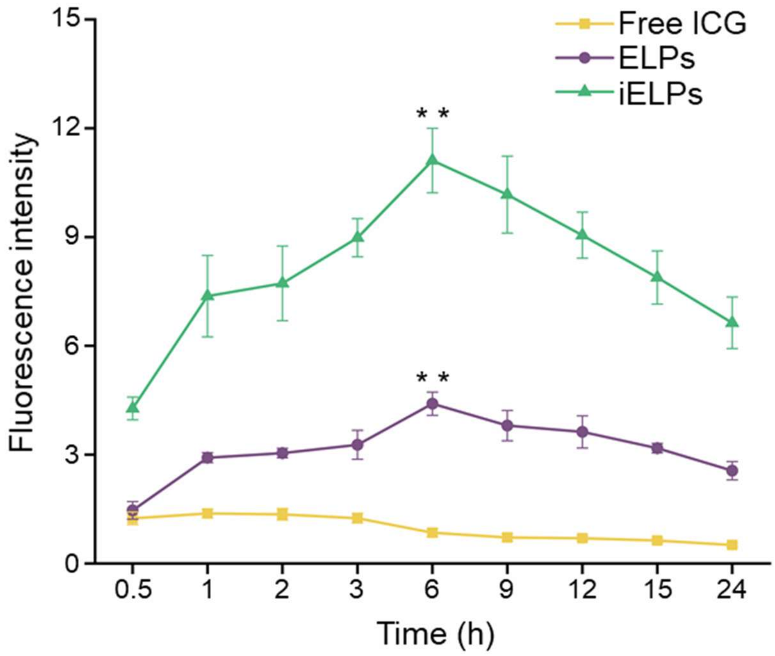

Figure S7. Time-dependent change in NIR fluorescence intensity of two symptomatic paws in CIA mice treated with free ICG, ELPs or iELPs (**P < 0.01).

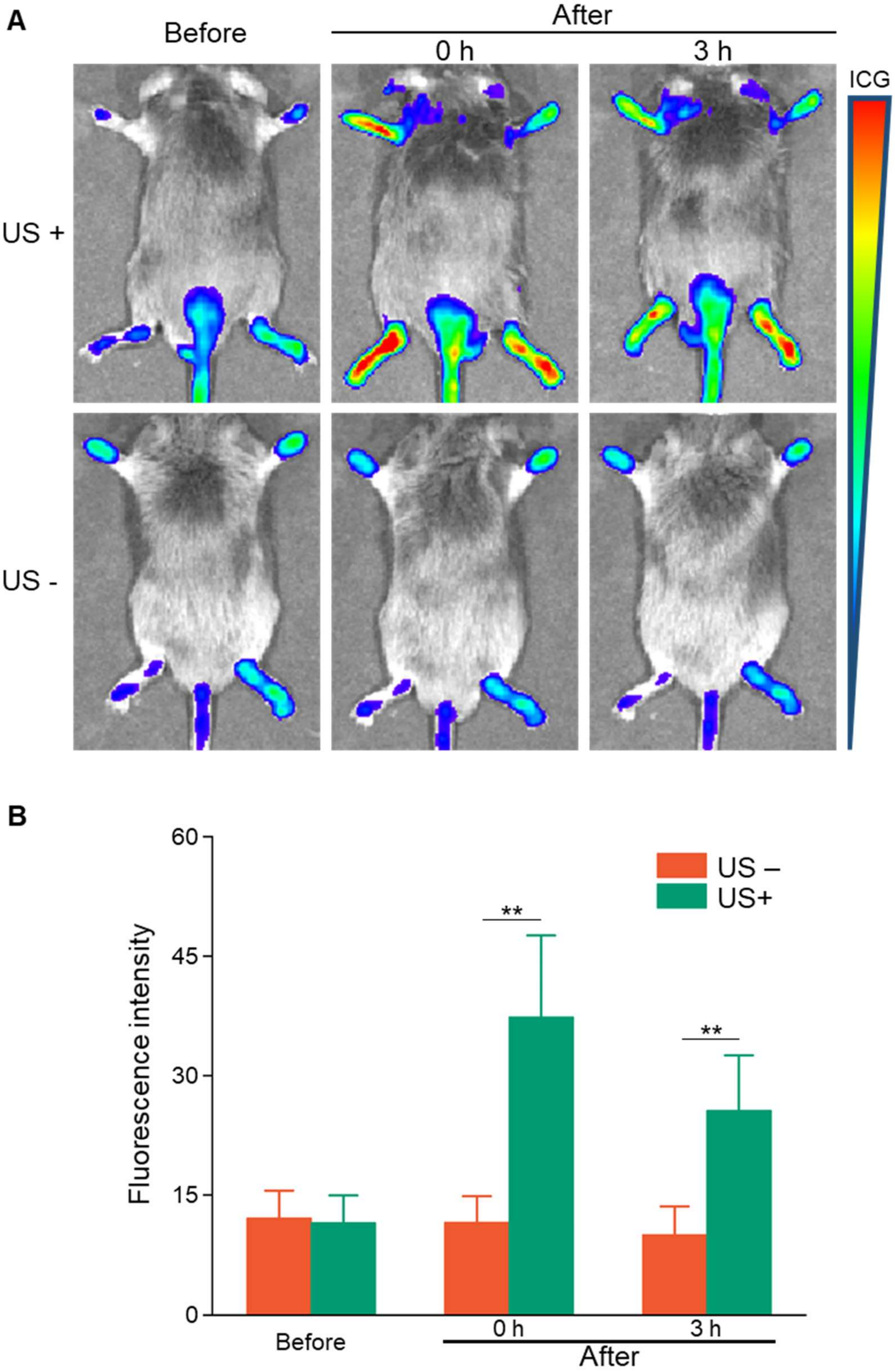

Figure S8. (A) NIR images of CIA mice treated with iELPs before and after ultrasonication. (B) Comparison of fluorescence intensities in the paws (** $P < 0.01$).

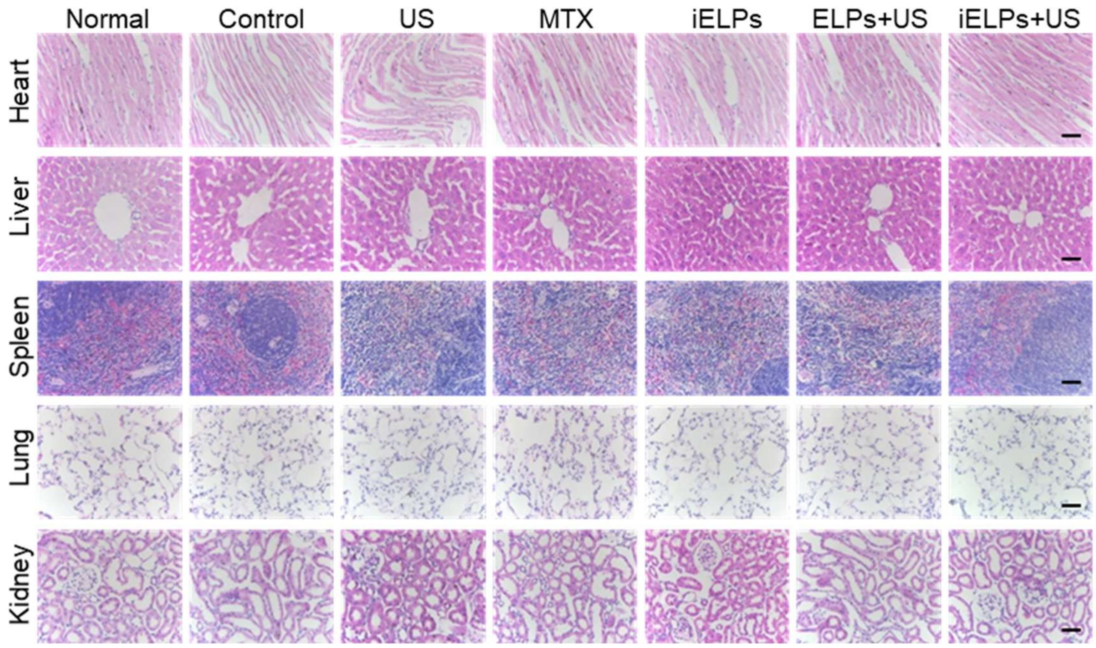

Figure S9. H&E stained images of major organs from the differentially-treated mice. (Scale bar = 50 μm.)
